# Public health use of HIV phylogenetic data in sub-Saharan Africa: ethical issues

**DOI:** 10.1136/bmjgh-2023-011884

**Published:** 2023-07-05

**Authors:** Euzebiusz Jamrozik, Nchangwi Syntia Munung, Lucie Abeler-Dorner, Michael Parker

**Affiliations:** 1 Ethox and the Wellcome Centre for Ethics and Humanities, University of Oxford, Oxford, UK; 2 Royal Melbourne Hospital Department of Medicine, University of Melbourne, Parkville, Victoria, Australia; 3 Monash Bioethics Centre, Monash University, Melbourne, Victoria, Australia; 4 Faculty of Health Sciences, University of Cape Town, Cape Town, South Africa; 5 Nuffield Department of Medicine, University of Oxford, Oxford, UK

**Keywords:** HIV, public health, health policy, epidemiology

## Abstract

Phylogenetic analyses of HIV are an increasingly accurate method of clarifying population-level patterns of transmission and linking individuals or groups with transmission events. Viral genetic data may be used by public health agencies to guide policy interventions focused on clusters of transmission or segments of the population in which transmission is concentrated. Analyses of HIV phylogenetics in high-income countries have often found that clusters of transmission play a significant role in HIV epidemics. In sub-Saharan Africa, HIV phylogenetic analyses to date suggest that clusters of transmission play a relatively minor role in local epidemics. Such analyses could nevertheless be used to guide priority setting and HIV public health programme design in Africa for sub-populations in which transmission events are more concentrated. Phylogenetic analysis raises ethical issues, in part due to the range of potential benefits and potential harms (ie, risks). Potential benefits include (1) improving knowledge of transmission patterns, (2) informing the design of focused public health interventions for subpopulations in which transmission is concentrated, (3) identifying and responding to clusters of transmission, (4) reducing stigma (in some cases) and (5) informing estimates of the (cost-)effectiveness of HIV treatment programmes. Potential harms include (1) privacy infringements, (2) increasing stigma (in some cases), (3) reducing trust in public health programmes, and (4) increased prosecution of legal cases where HIV transmission, homosexuality or sex work is criminalised. This paper provides analysis of relevant issues with a focus on sub-Saharan Africa in order to inform consultations regarding ethical best practice for HIV phylogenetics.

**SUMMARY BOX**

- Phylogenetic analyses have shown different patterns of HIV transmission in ‘generalised’ epidemics in sub-Saharan Africa as compared with ‘concentrated’ epidemics in high-income countries, where such data are increasingly used by public health agencies.
- This ethical analysis of HIV phylogenetics in Africa explores how the use of genomic data regarding patterns of HIV transmission might inform the ethical development of local public health policy, practice and research.
- Using phylogenetic data to inform the design of HIV diagnosis and treatment programmes for people more likely to be the source of transmission to others can produce greater public health benefits per individual treated, but these programmes must avoid exacerbating stigma and injustice.

## Introduction

Sequencing of the viral genome of HIV infections enables molecular and/or phylogenetic analyses of the relationships between viruses for public health and research purposes (table 1).^1 2^ This paper provides a preliminary analysis of ethical issues associated with uses of HIV phylogenetics in population health research and public health practice, with a focus on sub-Saharan African settings. We aim to contribute to ongoing consultations regarding appropriate governance policies for the use of HIV phylogenetics in public health contexts.

**Table 1:** Definitions of relevant terms

Phylogenetics	Analysis of the relationships between different samples of HIV including the creation of trees (phylogenies) that reconstruct historical patterns of HIV transmission in a population
Molecular epidemiology	The study of the distribution and/or transmission of different genotypes of HIV and other pathogens in human populations
Molecular source attribution	The use of phylogenetic analysis to identify individuals or groups as the likely source of transmission events and/or epidemics
Molecular surveillance	The use of genetic sequencing and/or phylogenetic analysis of HIV and other pathogens in public health surveillance
Public health surveillance	‘The continuous, systematic collection, analysis and interpretation of health-related data needed for the planning, implementation and evaluation of public health practice’^34^
Cluster detection and response	The use of sequencing data by public health agencies to identify and respond to clusters of related infections

Worldwide, there has been a growing interest in the role of phylogenetic analysis in public health responses, including during the COVID-19 pandemic. There have also been increasing concerns, including among people living with HIV, that such analyses may result in greater infringements of human rights.^1^ Sub-Saharan African datasets and phylogenetic expertise have been prominent in previous analyses of HIV^3 4^ and in the recent COVID-19 pandemic.^5^

Phylogenetic analyses can inform public health priority setting as well as the design and implementation of specific policy interventions. Although previous work in viral genomics examined relatively large viral genetic groups (eg, strains of HIV-1), recent developments in next generation (ie, high-resolution) HIV sequencing permit richer analyses of variations in viral genetic sequences within and between people living with HIV (at a much higher level of detail than analyses of HIV strains). These data can inform increasingly accurate assessments of, for example, (1) the dominant patterns of HIV transmission (eg, concentration of transmission in sub-populations or specific groups, the contribution of clusters to overall incidence, etc); (2) the effects of treatment on transmission (thereby improving estimates of the indirect benefits of treatment programs^6^; (3) the identification of individuals involved in transmission events (as either the source or the recipient of an infection); and (3) the directionality of transmission.

The use of phylogenetic approaches with data from next generation sequencing in public health research or surveillance (see table 1 for definitions of terms) can be used to clarify patterns of transmission at multiple levels, ranging from large subpopulations (eg, young men), to medium-sized groups (eg, people residing in a particular area), to small clusters of several individuals, or transmissions linked to one person (table 2). Similarly, public health interventions (eg, treatment programmes) can be guided by phylogenetic data at multiple levels, from designing treatment programmes for large groups to interventions focused on individual access to treatment.

**Table 2:** Levels of phylogenetic analysis by group size with example policy responses

Group size	Large segments of population	Identifiable groups	Large clusters (10+ people)	Small clusters (2–9 people)	Individuals
Examples of groups	Young adult males	MSM, PWID, residents of a local area, migrant populations	Several linked social networks	Small social networks	People identified as the source of multiple transmission events
Examples of policy response	Redesign HIV services for young men	Community engagement (with identifiable community)	Contact tracing, community engagement (with relevant networks)	Contact tracing and testing, informed by phylogeny	Directly observed therapy
Size of group identified	Large	↔	Medium	↔	Small
Identifiability of specific individuals	Minimal	↔	Moderate	↔	Significant
Cost of policy per additional person treated	Low	↔	Moderate	↔	High

MSM, men who have sex with men; PWID, people who inject drugs.

The use of HIV genomics in public health research and practice raises ethical issues, in part because phylogenetic analyses are associated with a range of potential benefits and harms (table 3). Ethical evaluations should arguably be informed by expected capacities for phylogenetic analyses as well as local HIV epidemiology, public health policy responses, local laws, and social norms.^7^ A particular ethical issue might become more (or less) important based on, for example, the degree of confidence with which phylogenetic data can link individuals and groups to HIV transmission events as well as how such data are used in policy and practice. Experience from the US and different European countries have shown that both real and perceived risks and benefits can shift greatly depending on who has access to the data, for example, if data collected for public health purposes are shared among government departments and/or law enforcement, and whether programmes were set up in consultation with people living with HIV. The following section summarises recent developments in the molecular epidemiology of HIV, phylogenetic technologies and technical capacity for genetic sequencing, with a focus on sub-Saharan African settings.

**Table 3:** Phylogenetic technical capacities and examples of associated benefits and harms

Phylogenetic technical capacities		Ethical issues	
Goal or purpose	Example	Examples of benefits	Examples of Harms
Identify transmission events	Identification of transmission pairs—two individuals likely involved in a transmission event	Improved access to care for those identified	Stigma, privacy infringements, reduced trust
Understand directionality	Identification of the sources and recipients of transmission events	Understand risk factors for transmission	Stigma, criminalisation, effects on employment and personal relationships
Clarify patterns of transmission	Identify concentrated transmission within groups or geographic areas	Improve access to care for identified subpopulations	Stigma, privacy infringements, reduced trust
Detect clusters of transmission	Detection of clusters of transmission	Inform cluster-focused public health responses	Privacy infringements, reduced trust
Estimate public health benefits	Determine effectiveness of interventions on transmission	Clarify (cost-) effectiveness	Privacy infringements

## Recent developments in the use of HIV phylogenetics for research and practice

There have recently been significant increases in sequencing capacity, including in sub-Saharan Africa, in part related to the COVID-19 pandemic.^5^ As such capacity has increased, so has awareness of phylogenetic sequencing among clinicians, public health practitioners and members of the public. Sequencing data are collected for different purposes in clinical, research and public health contexts and have been used in many countries for SARS-CoV-2 or for example during Ebola outbreaks. In sub-Saharan Africa, HIV sequencing has been used mostly in research contexts and to inform clinical drug resistance testing. The incorporation of HIV sequencing into public health programmes would require appropriate policies for data collection, use and linkage. These policies should be informed by scientific, technical and ethical considerations.

### Molecular epidemiology of HIV in sub-Saharan Africa

Phylogenetic data have enabled more accurate characterisation of the molecular epidemiology of HIV (ie, the distribution of different genetic lineages of HIV in human populations). The majority of phylogenetic analyses have been conducted in high-income settings, where epidemics tend to be ‘concentrated’ among certain groups or ‘key’ subpopulations such as men who have sex with men (MSM), people who inject drugs (PWID), etc. More recently, with wider access to sequencing technologies and relevant expertise, phylogenetic techniques have increasingly been used to understand transmission in low-income countries with large heterosexual epidemics, including in sub-Saharan Africa.^3 8 9^

It might have been expected that phylogenetic analyses in sub-Saharan Africa would identify significant clusters of HIV transmission within ‘generalised’ epidemics (A widely used threshold for a ‘generalised’ HIV epidemic is one where at least 1% of pregnant women are HIV positive). Such clusters might be amenable to highly focused public health responses similar to those used in high-income settings.^6 10^ However, as detailed below, phylogenetic analyses to date suggest that large high intensity clusters do not currently play a significant role in sub-Saharan African HIV epidemics, although these analyses nevertheless reveal patterns that might be used to inform high-level public health priority setting and overall HIV programme design (as opposed to highly focused responses to specific clusters).

Phylogenetic data from multiple sub-Saharan African contexts suggest that large clusters of transmission account for a minority of new infections. Although it remains possible that some (larger) clusters remain undetected, the available sub-Saharan African data suggest that most new HIV infections occur in long transmission chains where an infected person transmits HIV to 1 or 2 other people; large clusters of 10 or more people are extremely rare and linked transmission events may be spread out over long periods of time. Most infected people were found to not pass on the virus at all.^8 11 12^

Even if clusters of transmission are not a significant feature of current African epidemics, phylogenetic analysis has nevertheless clarified the segments of sub-Saharan African populations in which onward HIV transmission events may be more concentrated, for example, young men (aged around 30–35) and women (aged around 20–25).^13^ As opposed to high transmission in small clusters, the relative concentration of transmission in large segments of populations (involving millions of people) might warrant different public health policy responses.

Data demonstrating concentration of transmission, whether collected by researchers or public health agencies, could be used to improve access to treatment among relevant groups via (more) tailored public health services.^14^ Increasing treatment among those most likely to be the source of transmission will typically result in a larger average public health benefit per person treated (by reducing future transmission to others). Tailored diagnosis and treatment programmes could improve public health services without necessarily employing real-time molecular surveillance and/or responding to specific clusters of transmission. In any case, sufficient data privacy and protection from law enforcement would need to be guaranteed before implementation.

Molecular epidemiological analysis can also help to evaluate the benefits of policy interventions. Phylogenetic data demonstrated, for example, that wider implementation of universal test and treat policies in sub-Saharan Africa produced indirect benefits beyond treated individuals by reducing transmission to others.^8 15^

### Technical developments

Molecular epidemiology is a rapidly advancing area of research and public health practice. Key ongoing developments include (1) innovations in laboratory technology, for example, increases in the level of detail (or ‘depth’) of HIV sequencing; (2) increases in laboratory capacity, for example, next generation sequencing infrastructure producing rapid results, (3) increases in the size of phylogenetic databases, for example, the accumulation and/or linking of more data from larger numbers of individuals and populations; (4) innovations in analytic approaches, for example, novel applications of mathematical techniques to phylogenetic data; and (5) wider availability of expertise in the use of such techniques.

The accuracy of inferences regarding transmission of HIV from phylogenetic data is influenced by several factors including (1) depth of sequencing, (2) accuracy of sequencing (ie, the absence of erroneous or ambiguous sequencing reads), (3) population sampling, (ie, the degree to which a given phylogenetic dataset is representative of the population of people living with HIV); (4) availability of longitudinal data (eg, repeated sequencing of a particular person’s virus over time) and (5) the type of analysis used. As an example of recent results, the accuracy of identification of pairs of individuals involved in transmission events within relevant datasets has been reported to be over 95%.^16^

There may sometimes be a significant degree of uncertainty regarding assessments of direct transmission (eg, whether person A infected person B), for example, because simple HIV genetic analyses could not exclude other possibilities such as (1) reverse transmission (B infected A), (2) common source of infection (person C infected both A and B), or (3) unsampled intermediaries (A infected person D who then infected B). However, more recent analytic approaches can exclude these possibilities with a much higher degree of certainty, if deep sequencing data are available on a large fraction of people living with HIV.^17 18^ On the one hand, this higher degree of confidence in assessments of the details and direction of transmission raises concerns regarding harm to individuals identified as sources of transmission (eg, via stigmatisation or the use of such data in criminal investigations). On the other hand, these approaches can provide an even higher degree of confidence when ruling out particular individuals or groups as a source of transmission, which can help to reduce stigmatisation, for example, of migrant and/or underprivileged populations.^9 19^

### Policy

HIV public health policy responses are also evolving. In 2019, federal US public health agencies adopted the goal of eliminating local HIV transmission.^20^ Among other interventions, this involves enhanced cluster detection and response (CDR, see table 1), sometimes including phylogenetic analysis.^21^ This led to calls for a moratorium on HIV phylogenetic analyses in certain research or public health contexts.^22^ Such analyses can be controversial, in part because they may not involve informed consent for linkage or the ability to opt out. This has led to calls for limits to be imposed on public health use of molecular data, although such uses and/or limits have yet to be standardised.^22^ The question of whether data should be used for public health purposes without consent of the patients continues to divide opinion.^22^

Elimination of HIV transmission remains a more distant goal in sub-Saharan Africa than in North America. However, public health use of phylogenetic data will be increasingly feasible in the global South. While ethical uses of such data may produce significant public health benefits, misuse could erode trust in public health services.^23^ Without trust, people may be less willing to engage with testing (and treatment) for HIV. Among other things, this could undermine the potential benefits of phylogenetic surveillance, especially if the individuals who lose trust are also most likely to be the source of transmission events. Laws that criminalise HIV transmission and/or homosexuality may provide strong disincentives to participate in testing—especially if HIV genomic data could be used as evidence of sexual contact between individuals.^24^ Given such trade-offs between potential public health benefits and harms, ethical analyses should play a key role in the development of appropriate policy.

## Ethical issues

Phylogenetic analyses of HIV are associated with both potential benefits and potential harms (ie, risks). Determining ethically acceptable uses of HIV genomic data often turns on the balance of potential benefits and harms and the fair distribution of such outcomes, as well as other ethical considerations (eg, privacy). Below, we summarise relevant potential benefits and harms before discussion of how these can be evaluated in policy-making.

### Benefits

In addition to advancing scientific knowledge, HIV genomic analyses can be associated with individual benefits (eg, resistance mutation testing) as well as public health benefits. At the population level, molecular epidemiology research and/or public health surveillance can be used to clarify patterns of HIV transmission at multiple levels, ranging from large segments of the population to small social networks and individual transmission pairs (table 2).^9 25^ These data can be used to guide programmes or interventions aimed at producing public health benefits in several ways (table 3).

#### Improving HIV public health programme design

Identifying specific groups or subpopulations as more likely to be sources of transmission (than other subpopulations) can be a public health benefit via improved access to HIV diagnosis and treatment for these groups. This not only reduces the risk of AIDS among people living with HIV in these groups but also prevents new cases of HIV among their contacts. The number of new cases prevented will be proportionate to the likelihood that those who start treatment would otherwise have been the source of transmission to others. For example, sub-Saharan African phylogenetic data suggest that men aged around 30–35 are the source of a relatively large fraction of transmission events (per infected individual).^13^ One policy response would be to improve diagnosis and treatment services for this group to improve access to treatment. There might be a particularly strong ethical case for such policies if young men have been relatively neglected by treatment services and/or funding in the past and/or if those they are most likely to infect (eg, young women) are vulnerable in other ways.^26^

Phylogenetic analyses can also be used to identify clusters of transmission in social networks and/or specific individuals as the likely source of multiple transmission events (table 2).^25^ In principle, though not yet in practice, public health surveillance involving continuous collection of molecular data could guide cluster-based or individual-level interventions (eg, to improve access to treatment). The identification of small groups and individuals however carries an increased risk of harm and stigma (table 3). Public health programmes should, therefore, be extremely careful to avoid publishing data that might result in such harms.

#### Reducing stigma

Phylogenetic analyses can sometimes reduce stigma. For example, if there are rumours that a particular group of people within a community are the source of an HIV outbreak, phylogenetic analyses can be used to show that the group in question are either not over-represented in a database of transmission events, or that an outbreak is not in any way linked to the group in question. Making people aware that such rumours are not true could, in principle, help to reduce stigma. This approach has been used to show that a high prevalence of HIV in villages in the Lake Victoria region has not resulted in significant spread of HIV to surrounding communities.^9 27^

### Harms

Phylogenetic analyses of HIV can be associated with a range of harms, burdens, or infringements on privacy and other individual liberties.^28^ Such infringements are often associated with harms although they can also be morally problematic whether or not harm actually occurs.

#### Increasing stigma

The identification of individuals or groups as the source of HIV transmission can be associated with a significant increase in stigma. This can affect livelihoods, social standing, mental health and/or increase HIV transmission risks.^6^ Increasing awareness of the accuracy of molecular source attribution may undermine trust in public health (discussed below), which may in turn result in some people disengaging with further testing and treatment, potentially increasing risks of transmission.^1^

Stigma can affect very large groups of individuals, even comprising the population of nations or regions. For example, the widely criticised closure of international travel from sub-Saharan African countries on the discovery of the omicron variant of COVID-19 by local phylogenetics experts^5^ arguably involved the stigmatisation of countries based on (among other factors) the inaccurate view that the variant could be easily confined to those populations.

#### Undermining trust in public health

One concern regarding the use of HIV phylogenetics in public health practice is that some uses might normalise certain infringements of individual liberties and human rights (eg, to privacy), and/or result in a wider erosion of protections for people living with HIV.^1^ People living with HIV and their contacts need to be able to trust health services in order to engage with diagnosis and treatment programmes.

Violations of trust due to molecular public health surveillance might, therefore, worsen inequities in health and/or undermine efforts to reduce HIV transmission. Such concerns might arise when phylogenetic analyses accurately identify sources of transmission but also in cases where such attributions are erroneous. Researchers and public health agencies must, therefore, strive to maintain and increase trust, including by engaging with relevant communities and taking care to ensure ethically appropriate use of HIV phylogenetic data.

#### Infringements of privacy and other liberties

Highly focused interventions involving small numbers of individuals (eg, in clusters of transmission) potentially involve significant infringements of privacy and/or other liberties. Interventions focused on small numbers of people mean that these individuals can be more easily identified, which may result in stigma and/or reduced trust in public health.^23^

Infringements of privacy may also arise because phylogenetic relationships between viruses reveal information about, and relationships between, multiple people. For example, phylogenetic analysis of a new case might suggest that the person who was the likely source of transmission (eg, an older case) is no longer receiving highly active anti-retroviral treatment (HAART) or has developed resistance to treatment. It might also suggest direct (sexual) contact between two people resulting in HIV transmission.

Molecular analysis of HIV transmission might also infringe privacy by, for example, revealing infidelity and/or transmission between people of the same sex. This may result in particularly significant consequences if data are shared with law enforcement in jurisdictions where homosexuality is criminalised. In research contexts, many of these risks can be mitigated by appropriate data protections, including storage of data in jurisdictions without such laws (although this may conflict with norms regarding data sovereignty). In contrast, public health agencies who hold HIV genomic data may be less able to mitigate such risks, for example, where there is an expectation of data sharing between public healt and other government agencies, including law enforcement.

In principle, phylogenetic data showing multiple transmissions from a particular individual could be used to enrol such individuals in directly observed therapy, where a health worker checks individual medication use on a regular basis^29^—a strategy commonly employed for tuberculosis. However, this involves significant liberty infringement, can increase stigma, and might be particularly problematic where HIV transmission is criminalised.

#### Prosecution of criminal cases

Where HIV transmission or homosexuality is criminalised, phylogenetic analyses may be used to inform legal judgements about whether a particular individual was the source of transmission or whether two individuals of the same sex were involved in a transmission event.^17 18^ Such analyses have been used as evidence to substantiate (or refute) criminal charges of HIV transmission^30^ and could in principle be used as evidence of sexual contact.

Although some low-income and middle-income countries, including in sub-Saharan Africa, have laws that explicitly criminalise HIV, criminal convictions are more common (per diagnosed cases of HIV) in high-income countries than in low-income countries.^31^ However, this could change depending on local laws,^32 33^ especially where these include punishments such as imprisonment or the death penalty, and/or where increasingly available HIV genomic data could be misused. Health agencies and legislators should therefore consider the extent to which even legally permissible uses of scientific data might worsen the stigmatisation of people living with HIV and other infectious diseases.

Similarly, there is a widespread consensus within public health ethics that public health agencies should not share data for any purpose (including criminal cases) other than for interventions aiming to produce public health benefits.^34^ One reason why criminalisation of transmission might be harmful to public health more broadly is that fear of such consequences might lead individuals to avoid diagnosis. The consequences of criminalisation might, therefore, be a wider public health harm due given the potential to undermine successful responses to HIV epidemics.^35^

## Discussion

Most work on the ethics of phylogenetics has focused on high-income settings,^36^ in part because of greater use of these approaches in high-income countries. Other scholars have added new perspectives and summarised qualitative data regarding ethical issues in sub-Saharan African contexts.^37–40^ This paper provides an up-to-date analysis of ethical issues in such contexts, informed by recent scientific advances. Increasing use of molecular data in sub-Saharan Africa has shown that phylogenetically linked *clusters* of transmission appear to play a relatively minor role in generalised HIV epidemics. However, they have also shown patterns of transmission that might have (other) implications for HIV public health programme design. Below, we provide a preliminary ethical analysis of molecular public health responses, with the goal of stimulating further discussion and consultation that could form the basis of ethical frameworks for best practice.

### Risk–benefit assessments

There is widespread agreement that the benefits of research and/or public health policy should outweigh associated risks (ie, potential harms) and/or liberty infringements.^36^ However, risk–benefit assessments are often made more complex by (1) lack of quantification of relevant benefits or risks, (2) differences in kind (as well as in quantity) between multiple relevant benefits or risks (meaning that even if they were quantified they could not be put into a simple mathematical equation), (3) conflicts between incommensurable values such as health, fairness and freedom (or utility, equality and liberty) and/or (4) differences in the cultural, social and legal context in which specific epidemics or interventions take place. Below, we discuss how ethical analysis can help to evaluate when the expected benefits of more focused programmes based on molecular surveillance may be considered to outweigh potential costs and risks and vice versa.

### HIV programme design informed by phylogenetic data

Phylogenetic data can be used to inform (1) public health priority setting, (2) the design of specific HIV programmes and/or (3) estimates of the benefits or cost-effectiveness of HIV treatment interventions (by evaluating the indirect benefits of treatment in reducing transmission of HIV).

Given phylogenetic data in sub-Saharan Africa showing that men aged in their 30s are more likely to be the source of transmission of HIV in generalised epidemics (than older males or age-matched females),^13^ there would be greater public health benefits of linking young and middle-aged men to HIV care (per individual) than other population groups. Such data might in general support reallocation of HIV programme resources towards sources of transmission and away from the recipients of transmission events, assuming resources are relatively fixed. Such reallocation would result in a greater reduction in incidence of new infections (eg, among groups more likely to be infected) per individual treated. However, such changes raise complex ethical questions because groups more likely to be recipients of HIV infection (eg, young women) might also be considered to warrant relatively greater resources in part because of structural and/or socioeconomic factors that prevent them from being able to access other forms of care.

Similarly, insofar as phylogenetic data from sub-Saharan African epidemics shows that transmission is more evenly spread across the population (compared with highly clustered transmission in ‘concentrated’ high-income country epidemics) one response might be to reallocate HIV programme resources from certain groups to other segments of the population. However, it might be argued on grounds of justice or fairness that certain groups warrant greater resources, whether or not they are over-represented as sources of HIV transmission. For example, sex workers or men who have sex with men might warrant greater resources insofar as they are socially or economically marginalised, regardless of whether such groups are disproportionately represented in transmission data. Alternatively, it might be argued that it is the very success of HIV programmes tailored for these groups that prevents them from being disproportionately involved in transmission. The latter claim could be tested in molecular epidemiological research.

In contrast, there might be cases where justice-based claims and data from molecular epidemiology are aligned, for example, where a marginalised group is shown to be disproportionately involved in HIV transmission (as in ‘key’ populations in high-income countries). In such cases, there would be an especially strong ethical rationale (on multiple grounds) to improve the design of, or access to, HIV public health programmes for these groups.

### Data protections in clinical, research and public health contexts

Personal data in clinical and research contexts are governed by well-established ethics norms and protected by multiple safeguards. More recently, phylogenetic data have increasingly been used in public health responses to HIV in high-income countries.^1 23^ Molecular data were also used in near real time during the recent COVID-19 pandemic, including in sub-Saharan Africa.^5^ The ethical and legal norms that should govern public health uses of sensitive data remain a matter of debate in public health ethics.^34^

**Figure 1:**
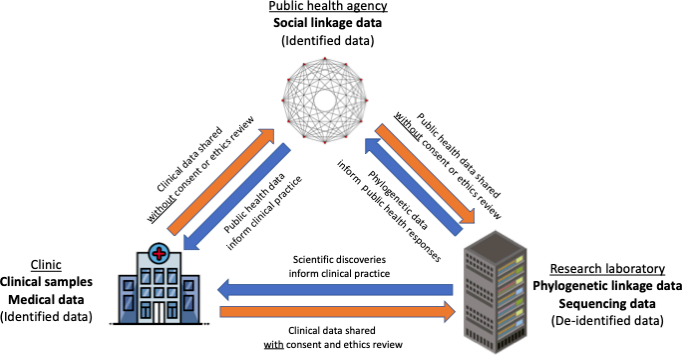
Data sharing between clinical, public health and research settings.

Among other differences from research settings, public health uses of data are typically not subject to individual informed consent requirements (figure 1).^34 41^ The reporting and notification of diagnoses of infectious diseases to public health agencies is a well-recognised exception to patient confidentiality. This exception is widely considered to be justified on the ethical grounds of protecting public health via knowledge of the prevalence and/or transmission of an infectious disease, which can be used to plan and fund services for that infection, including specific interventions for higher risk social networks, specific infected individuals, and/or their contacts.^34 42^ However, public health use of HIV sequencing data without consent or the ability to opt out of genomic linkage has also raised controversy, especially in the USA.^1 43^ Health agencies in all countries, including sub-Saharan Africa, should strive to use phylogenetic data for the common good, without undermining public trust.

Unlike phylogenetic research, which typically uses de-identified data, public health programmes may sometimes link phylogenetic data with identifying metadata (eg, name, date of birth, address). This might in principle help to increase public health benefits by enhancing existing contact tracing and/or CDR activities, however, such uses represent a significant risk to privacy, raising questions about the conditions under which the benefits outweigh privacy infringements and related risks. Such questions should also be evaluated in different local contexts, including in sub-Saharan African communities.

Privacy infringements can be reduced by setting appropriate limits on data sharing. For example, it is often unnecessary for clinicians or public health agencies to have access to detailed viral genetic sequences to help patients and protect public health. Likewise, it is often unnecessary for researchers to have access to highly identifiable information to study viral evolution. Therefore, there will rarely, if ever, be a justification for clinical, research and public health databases to be (fully) linked. Similarly, data protection policies should also prevent the sharing of data collected for health purposes with police and other agencies involved in criminal prosecution.^34^

### Cluster detection and response

HIV CDR requires active surveillance and contact tracing by public health authorities. While it does not necessarily require molecular surveillance, phylogenetic analysis might nevertheless make contact tracing more efficient by providing additional evidence of genetic linkage of cases within a specific transmission network. Conversely, analyses might show that a case with epidemiological links to a cluster nevertheless acquired infection elsewhere. In any case, these are resource-intensive activities used primarily in high-income settings and the benefits of CDR informed by phylogenetic analysis have not been quantified.^44^

Cluster responses are unlikely to be a high priority in most sub-Saharan African settings given that (1) data suggest that clusters currently play at most a minor role in generalised HIV epidemics, (2) public health resources are limited and (3) CDR might erode trust among key subpopulations^1 23^. In future, as more people living with HIV are established on treatment, a greater fraction of new infections may be shown to arise in clusters and/or from people linked by molecular surveillance with multiple transmission events. The prospect of such an epidemiological transition might prompt consideration of the ethical acceptability of molecular CDR in sub-Saharan Africa.

## Conclusions

Phylogenetic analysis of HIV has revealed important transmission dynamics that may help to inform public health practice and, in some cases, increase the benefits and/or cost-effectiveness of HIV diagnosis and treatment programmes. Given increasing availability of HIV sequencing data phylogenetic expertise in sub-Saharan Africa, such data will likely inform future public health policy and practice.

This project is intended as to inform future consultations aimed at developing frameworks for ethical best practice for HIV phylogenetics in sub-Saharan Africa. Such frameworks should also be informed by evolving evidence regarding transmission patterns which, as we have highlighted here, may not always resemble those in high-income countries. Such frameworks may draw on existing ethical frameworks for public health or human genetics and/or on African philosophical traditions.^34 36 45 46^ Policy development should also be informed by input from a wide range of stakeholders and communities, including people living with and/or at risk of HIV. Finally, policy should aim to be sensitive to community norms while also promoting more universal ethical commitments to improve health and avoid stigmatising people living with infectious diseases.

## Data Availability

No data were generated for this research.
